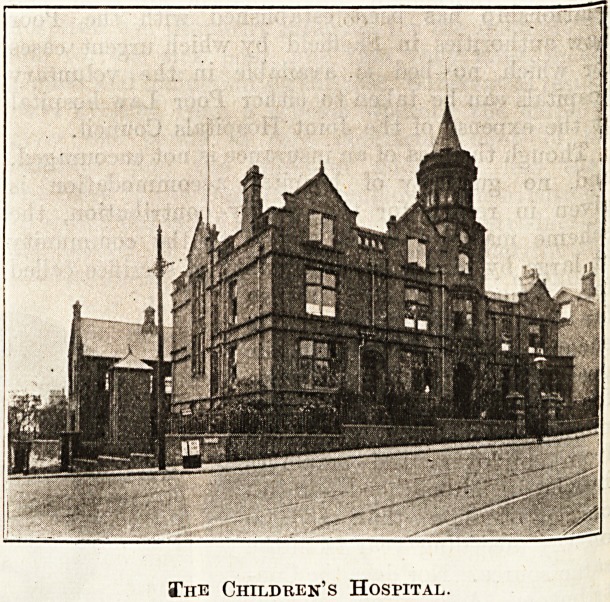# British Hospital Association

**Published:** 1923-06

**Authors:** 


### BRITISH HOSPITALS' ASSOCIATION.

### THE SHEFFIELD CONFERENCE.

As we announced last month, the annual conference of the British Hospitals' Association is to be held at Sheffield on Thursday and Friday, May 31 and June 1, under the presidency of the Hon. Sir Arthur Stanley, G.B.E. On the first day an address will be given by Mr. H. J. Waring, F.R.C.S., Vice-Chancellor of London University (Hon. Surgeon, St. Bartholomew's Hospital), on "The Future of Voluntary Hospitals from a Medical Viewpoint." In the afternoon an address on "Modern Hospital Administration," by Dr. H. L. Eason, C.B., Superintendent of Guy's Hospital, will be followed by a discussion, to be opened by Dr. S. S. Goldwater (Mount Sinai Hospital, New York). A reception at the Town Hall by the Lord Mayor of Sheffield and Sir Henry Hadow, Vice-Chancellor of Sheffield University and Chairman of the Sheffield Joint Hospital Council, will follow, and in the evening there will be an official dinner to the delegates, when the Chairman will be Sir Henry Hadow. The following day at 10 a.m. a business meeting to elect honorary officers and Council and to fix time and place of the fourteenth annual conference will be held, followed by a statement by Sir Napier Burnett on "Hospital Finance." Lord Hambleden will then speak on "Hospital Finance in its Relation to Approved Societies."

Mr. H. J. Waring, F.R.C.S.. Vice-Chancellor of London University.

While its most ardent supporter would not contend that the Sheffield Hospital system is either ideal in principle or thoroughly adequate in operation, it cannot be denied that it is progressing on sound business-like lines, and that even in these days of industrial instability its future may be viewed with a certain measure of hope and satisfaction. A new factor has come into play, and whether at bench or in Board-room a "hospital conscience" has been born, and with the steady expansion and development of that conscience has grown a spirit of civic beneficence which, in turn, insists that if the welfare of voluntary medical institutions is equally the charge of master and employé, so may they jointly claim at least a voice in management policy, and, as a necessary corollary, expect to bear a share of management responsibility. To-day Sheffield's five voluntary hospitals are united in their appeal, are unhampered by redundant effort, and work to a common end. Their position would have been unhappy indeed if, with a shrinkage of private subscriptions and an irreducible annual expenditure of £120,000, the hospital authorities had not combined three years ago to meet the dangers threatened by declining trade and unprecedented unemployment.

Mr. H. L. Eason, C.B., C.M.G., M.D.

Under the new policy of democratic control devised by the Joint Hospitals Council, virtually every force in the city's civic, educational, industrial and commercial life, irrespective of social considerations, has been harnessed to ensure active recognition of the fact (and its accompanying obligation) that the hospitals are primarily intended for the unemployed and for the sick poor, and that they must secure regular and unfluctuating support. Substantial results have accrued from a scheme whereby the employé gives one penny in each complete pound of his earnings, and the employer adds not less than one-third to the total of his employés' contributions. In return unlimited numbers of letters of introduction are placed at the disposal of each contributing establishment, to be used only for the workmen and their dependents. Hospital treatment is promised only so far as beds are available, and medical urgency is the only condition of admission. Committees have also been formed in municipal wards and neighbouring parishes so that shop-keepers, small

Alderman Charles Simpson. Chairman of Sheffield Voluntary Hospitals Committee manufacturers, and workers in minor establishments may have an opportunity of participating.

Managements and workmen of firms outside the city putting the penny-in-the-pound fund into operation are encouraged to distribute contributions proportionately to all hospitals serving the contributors and their families, whether in the city itself or in such adjacent areas as Barnsley, Chesterfield, Doncaster, Mansfield, Mexborough, Rotherham, Worksop, &c. The problem of securing financial support from outside districts of the West Riding, North Derbyshire, and North Nottinghamshire, from which in-patients are drawn, is gradually being solved. The co-operation of residents in those areas is being obtained, and it is hoped to reach a practical understanding with the boards of management of the county and cottage hospitals in those districts. Further, a working relationship has been established with the Poor Law authorities in Sheffield by which urgent cases for which no bed is available in the voluntary hospitals can be taken to either Poor Law hospital at the expense of the Joint Hospitals Council.

Mr. H. Wade Deacon, C.B.E.

Though the idea of an insurance is not encouraged, and no guaranty of hospital accommodation is given in return for the weekly contribution, the scheme makes a direct appeal to the community at large by reason of the equality of sacrifice called for. Smooth working has so far been secured, and it is encouraging to note that the possibilities offer ample scope for development. Last year more than £60,000 was raised from employers and employés in the neighbourhood—an increase of more than £35,000 on any preceding year from the same source. Nor does the Contributors' Association current membership of 154,000 represent the possible apex. A quarter of a million members is aimed at, and the organisers are confident that during this year £100,000 will result from the scheme. The Contributors' Association has been proportionately recruited from delegates of all the contributing establishments. Meetings are held four times a year to discuss hospitals' problems generally, and interest is thus maintained in the progress of the city's medical institutions.

Mr. S. R. Lamb, Secretary of the Sheffield Joint Hospitals Council.

The scheme of service does not stop there. Within financial limits it is proposed to keep trace of patients after they leave the hospital. An arrangement has recently been entered into with the Queen Victoria Nursing Association whereby any patient may, on application to the Joint Hospitals Council, be visited by a qualified nurse and a report given as to any after-care or convalescent treatment that may be needed. A comprehensive scheme of convalescent treatment embraces not only those who have been in hospital but those who, by preventive measures, may be saved from entering it. Any contributor or dependent, on the recommendation of a Works Hospital Committee and family doctor, may have choice of convalescent treatment from more than 40 institutions throughout the country, and be provided with return rail fare. Though all these facilities are now available the importance of the adage that prevention is better than cure has not been overlooked. At the contributors' request eight health lectures were given last year, at which some of the greatest authorities in the country dealt with various aspects of public and individual health problems. So successful was the experiment—the total attendance exceeded 10,000—that it is to be repeated during the winter of 1923.

Mr. J. Courtenay Buchanan, C.B.E., Hon. Sec. of the Hospitals Association.

It is recognised that substantial leeway has to be made good in hospital reconstruction. Those responsible are determined to remedy the tragic state of affairs under which the city's present waiting list falls only 50 short of 3,000. An agreed united policy is being formulated so that there shall be no duplication in the forward movement. Hospital accommodation is to be increased soon by 150 beds, and more adequate and generous facilities are to be afforded by the creation of a special fund for bringing patients to hospital and transporting them to out-patient departments.

With heavy responsibilities to meet and big problems to solve the hospital authorities are not depressed by the current heavy debt of £80,000. The accumulation of years, this debt has already been reduced from £116,000 in little more than 12 months, notwithstanding that only as recently as 1921 the year's revenue of the two general infirmaries was £40,000 less than their expenditure. In this difficult process of reconstruction the authorities are indebted to and encouraged by the splendid co-operation of all sections of the community. Particular mention may be made of Alderman Charles Simpson, Deputy Lord Mayor, and Chairman of the Sheffield Voluntary Hospitals' Committee; Councillor M. Humberstone, President of the Sheffield and District Hospital Contributors' Association; the presiding genius of the Sheffield Joint Hospitals' Council, Sir Henry Hadow, C.B.E., Vice-Chancellor of Sheffield University; and the present Master Cutler, Mr. R. W. Matthews, an indefatigable worker on behalf of the city's medical institutions, a prominent member of the Joint Hospitals' Council, and Vice-Chairman of the Royal Infirmary Board.

Sheffield Royal Hospital.

Sheffield Royal Infirmary.

The Jessop Hospital for Women.

The Children's Hospital.

---

The War Memorial Hospital Fund, Hastings, has now closed, having reached the sum of £62,000 asked for when it was started in 1919. The East Sussex Hospital moved to its new premises in the middle of May.

## Figures and Tables

**Figure f1:**
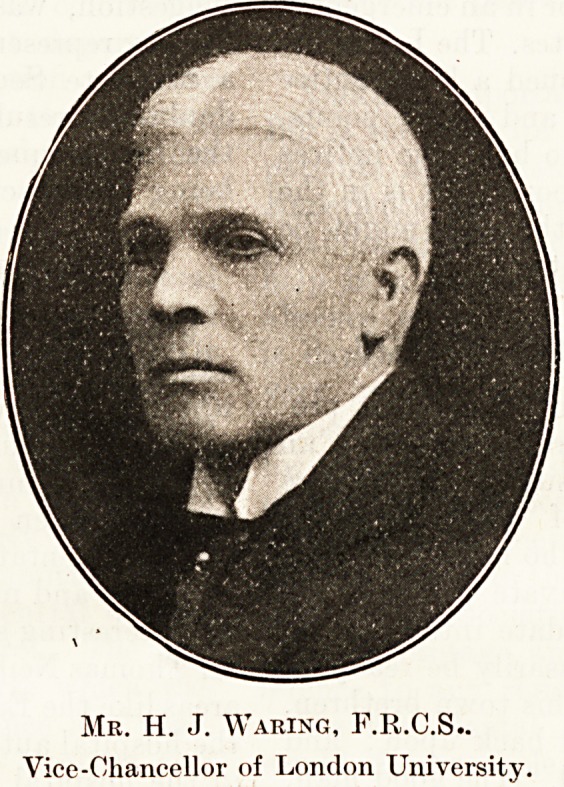


**Figure f2:**
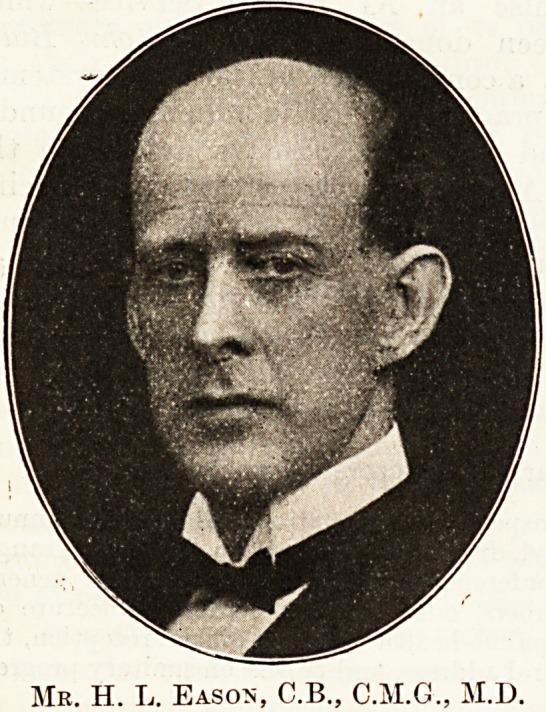


**Figure f3:**
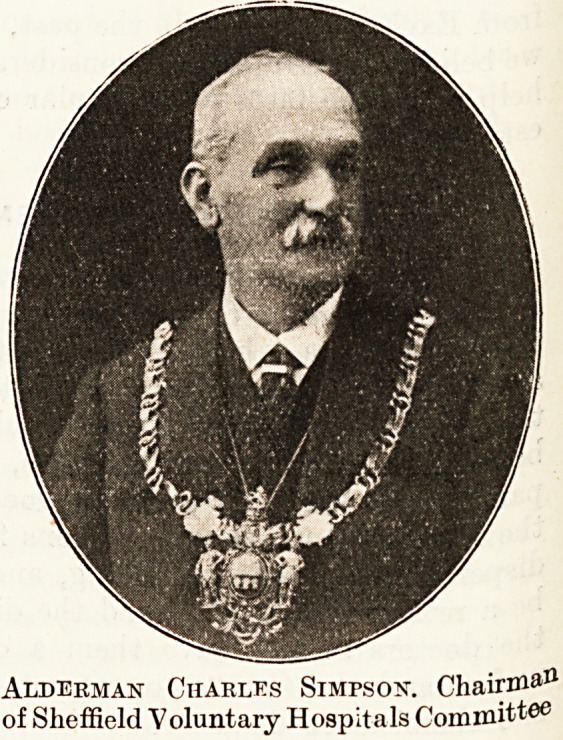


**Figure f4:**
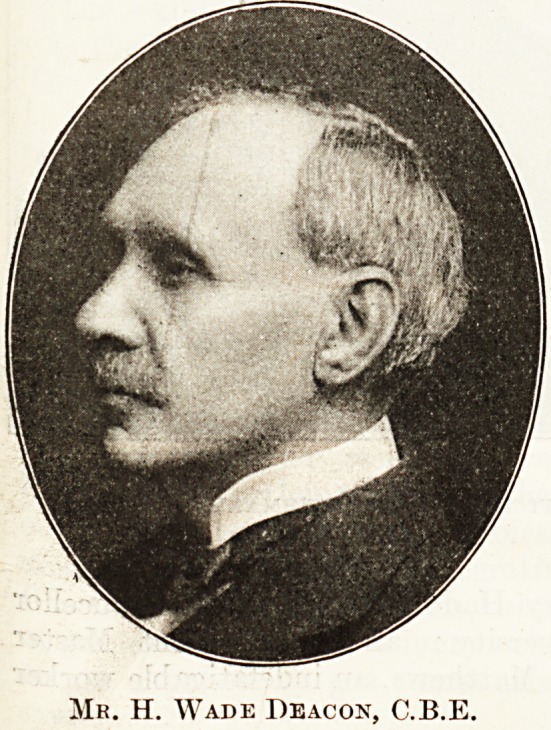


**Figure f5:**
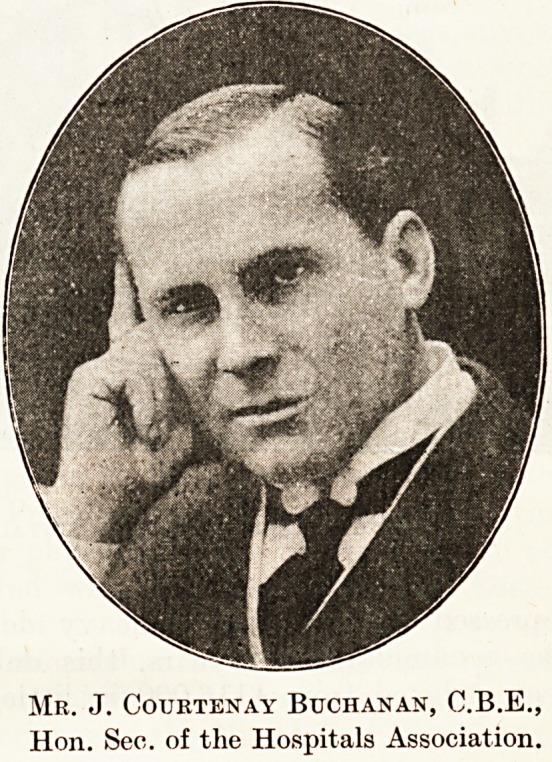


**Figure f6:**
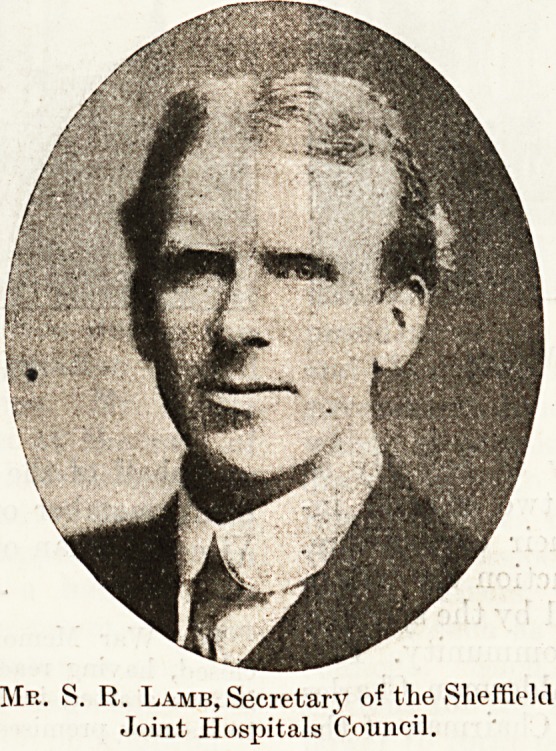


**Figure f7:**
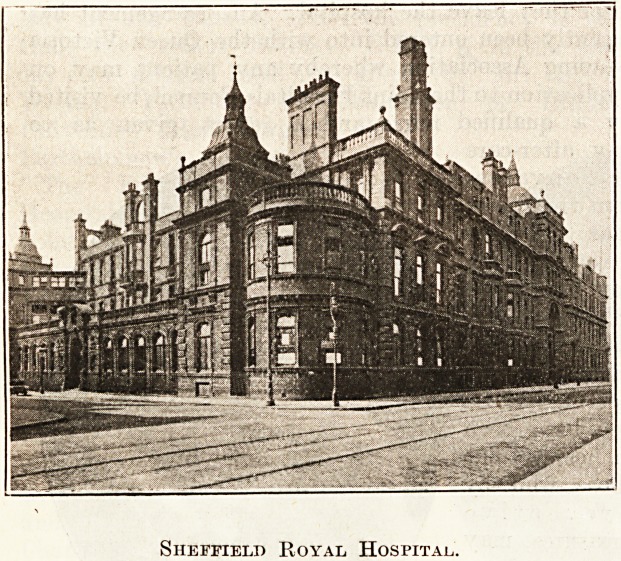


**Figure f8:**
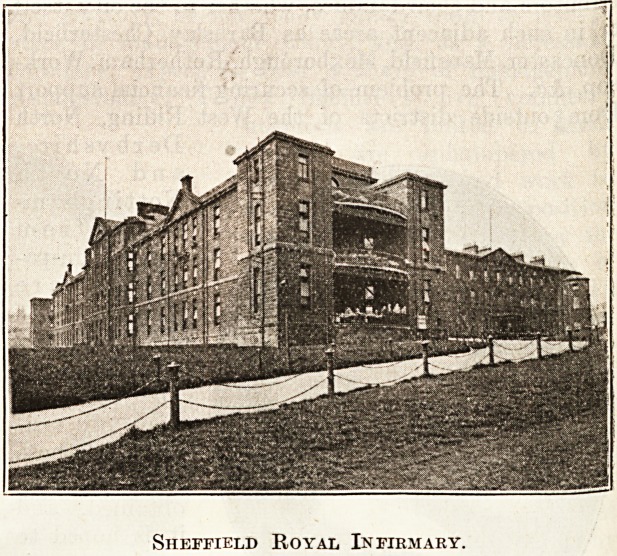


**Figure f9:**
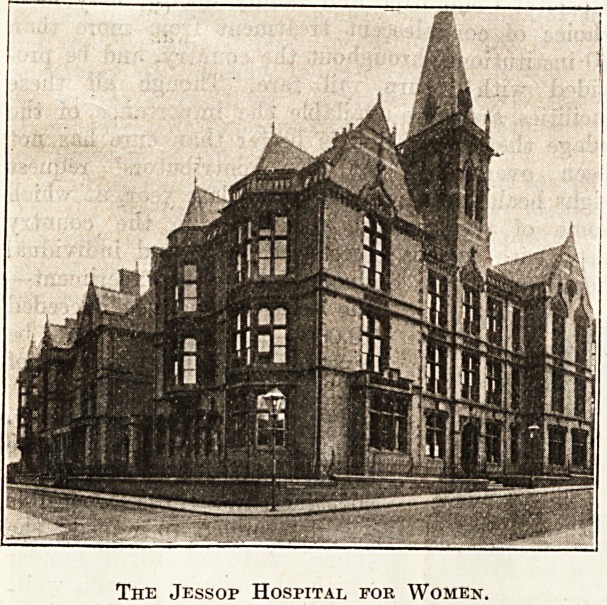


**Figure f10:**